# Biology and Health Inequality

**DOI:** 10.1371/journal.pbio.0050267

**Published:** 2007-10-23

**Authors:** Eric Brunner

## Abstract

Intriguing parallels between civil servant and nonhuman primate hierarchies suggest that highly stratified societies foster health inequalities. Determining how social differences translate into chronic disease remains a challenge, but neuroendocrine pathways appear to play a role.

Despite continued growth in the global economy, good health remains elusive for a large proportion of the world's population. Health inequalities between rich and poor countries are based on historical patterns of development and exploitation that leave millions of people starved of basic material and social amenities. In those rich countries where absolute poverty is largely absent, continuing health inequality is more difficult to understand. The discipline of biology has an important contribution to make in this regard. Studying the pathways responsible for translating social differences into biological differences and chronic disease is an intrinsically challenging pursuit. It is also an ethically compelling application of life science.

One of the root observations leading to the work I will describe arose from the first Whitehall study, which analyzed cardiovascular disease among 17,530 civil servants working in London 30 years ago [1]. In this population of middle-aged men, all of whom were wage-earners in stable jobs, there was an inverse relationship between social status and coronary heart disease (CHD) mortality: the lower the employment grade, the higher the risk of death. A ten-year follow-up of the cohort showed that there was a steep inverse relation between grade of employment and death from all causes—CHD and noncoronary causes [2]. The relative risk of death due to CHD was 2.2 in clerical compared with senior administrative staff, and 1.6 for those in the intermediate professional and executive grade. This working community shared a benign environment at the time of the baseline screening examination in 1968, important features of which included growing prosperity, the welfare state, and free health care. Nevertheless, a health hierarchy persisted that produced a greater than 2-fold difference in mortality, even across social strata limited to men in secure, non-manual employment. Mortality gradients in the study reflected those in United Kingdom social class mortality data in the 1970s, which continue today.

The then-novel technique of multivariate modeling shed light on some of the causes of the inverse social gradient in CHD in the first Whitehall study and helped to shape the future research agenda. When conventional coronary risk factors—such as smoking, serum cholesterol, and blood pressure—were controlled for one-third of the gradient was explained. Importantly, serum cholesterol levels were slightly higher in men in the higher employment grades [1]. The distribution of serum cholesterol in a population is a key determinant of its background level of risk, but it did not contribute to the social gradient. The implication was that the specificity of the biological factors lying behind the mostly unexplained disease gradient might help to identify the mechanisms responsible for it.

The discovery of stepwise rather than threshold nature of these health inequalities captured funding interest on both sides of the Atlantic. A second large cohort of civil servants was set up so researchers could study inequalities in health functioning and morbidity as well as mortality and explore the psychosocial, behavioral, and biological pathways involved [3]. The Whitehall II study recruited 6,895 men and 3,413 women, 35–55 years of age, with an initial questionnaire and biomedical examination in 1985–1988. Individuals have been followed through regular contacts, medical records, cancer registrations, and death certificates for over 20 years. There have been 737 (7.2%) deaths in the cohort to July 2006. The choice of civil servants is, to an extent, for convenience, because it is comparatively easy to keep track of participants. We are also studying a stratified institution within which men and women are subjected to an imposed work hierarchy—an illuminating population to study.

We observed many health disparities that are important from a public health perspective, meaning that they account for a substantial proportion of potentially preventable mortality and morbidity in the populations of rich countries as well as in the Whitehall II population. All-cause and cardiovascular mortality both follow inverse gradients, but cancer mortality does not. Incident type 2 diabetes and CHD follow inverse gradients. In terms of quality of life, there are social gradients in depression and physical functioning (based on self-report, multi-item questionnaires), which emphasize that clinical disease can be viewed as the tip of an iceberg of ill health. Positive health and well-being are not restricted to those at or near the top of the hierarchy, but the nearer an individual is to the top, the more likely he or she is to be in good health.

A biological pointer to the pathways responsible for the inverse social gradient in CHD emerged from our attempt to improve on the explanation provided by conventional risk factors in the first Whitehall study. We showed that there was a steep inverse social gradient in a cluster of risk factors known as metabolic syndrome (Figure 1) [4]. Metabolic syndrome has predictive power for CHD events in addition to that of its individual components. In men, prevalence of metabolic syndrome in the lowest employment grade was more than double that in the highest, and in women, prevalence differed more than 4-fold. The core features of metabolic syndrome are an abdominal distribution of obesity (which is associated with insulin resistance and hypertension), raised serum triglyceride, low high-density lipoprotein (HDL) cholesterol levels, glucose intolerance, and raised blood pressure. We found in explanatory models that behavioral factors (reported smoking status, alcohol consumption, and physical activity level) and general obesity (body mass index) accounted for one-third of social difference in metabolic syndrome prevalence in men, and only one-tenth in women.

**Figure 1:**
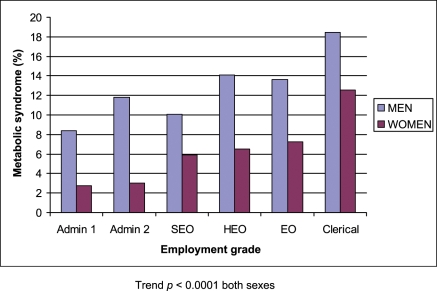
Prevalence of Metabolic Syndrome (Adult Treatment Panel III Definition) by Civil Service Employment Grade in the Whitehall II Study 5,382 men and 2,389 women aged 39–63. Trend test from logistic model adjusted for age and ethnicity. Admin 1, highest level administrators, e.g., permanent secretary; SEO, senior executive officer; HEO, higher executive officer; EO, executive officer; Clerical, clerical and office support staff.

That behavior-related factors did not provide a full explanation for the social gradient in metabolic syndrome is consistent with the operation of direct psychosocial and neuroendocrine pathways linking lower social status to CHD risk. The hypotheses we are testing concern the biological response of the individual to the social environment, drawing an analogy between low social status and chronic low-level stress exposure [5,6]. “Stress”—one of the most ambiguous words in the language—here refers to the psychosocial hypothesis. This hypothesis builds on the observation that the social environment has important psychological components that may influence health. Not only does the social hierarchy influence access to material resources, it also shapes the availability of psychological resources. Beliefs and emotions are socially patterned, and the regularity of such social differences across place and time suggests they may contribute to social differences in mental and physical health [7]. The idea is intrinsic to a variety of familiar psychosocial concepts including power, control, demands, and reciprocity (effort and reward). These and other related concepts such as gender and racial discrimination, justice, fairness, and autonomy are increasingly being studied with respect to their impact on a variety of biological factors and health outcomes in the interdisciplinary field of social epidemiology.

In contrast to this social “embodiment” perspective, it has been argued that biological integrity, in the form of inherited advantage, is the real cause of social inequalities in health. Cognitive function is a high-level expression of this property, and analysis of the changes in test scores over the first ten years of life in a population-based study (the 1970 Birth Cohort) emphasizes the importance of environmental influences. Cognitive performance was first measured at 22 months of age. Children who ranked low at baseline rose through the ranks if their parents were affluent, and conversely, children who ranked high at baseline fell back if their parents were poor. By age seven, the two groups had crossed over in the rankings, whereas children who had both high initial test scores and high social class parents remained near the top of the ranking, and vice versa [8]. These and numerous other findings suggest for the majority of those born without congenital disease, and regardless of the endowment of health capital, a poor early socioeconomic environment is likely to undermine development and later health prospects. Head Start and Sure Start are national programs in the United States and the United Kingdom designed specifically to interrupt the intergenerational cycle of deprivation.

The integration of disciplines in our work is challenging in an academic world dominated by a fierce reductionism. There is, however, a noble pedigree to the perspective, reaching back to Claude Bernard, Walter Cannon, and others who laid the foundations of physiology and systems biology. Central concepts include autonomic and neuroendocrine regulation based on interlocking feedback mechanisms, stress reactivity in the guise of the fight-or-flight response, and the limits of stability in organ systems (Box 1). More recently, Bruce McEwen and others extended the principle of homeostasis to include processes leading to disease with the allostatic load hypothesis [9]. While allostasis, the process of achieving stability (or homeostasis) through change, is critical to survival, a chronic response to stress may carry a cost (the allostatic load). The price of adaptation to external stress may be an adverse change in homeostatic systems, for example, pushing the individual toward a diabetic state.

A serendipitous connection with the work of Stanford neuroscientist Robert Sapolsky reinforced our motivation to pursue work on social status and metabolic syndrome. His studies of the olive baboon hierarchy in the Serengeti revealed remarkable parallels with our findings in London. Baboons live in a benign environment where food is plentiful, infant mortality is low, and predators are scarce. Social rank is a preoccupation among males. Just as in male civil servants, total and low-density lipoprotein (LDL) cholesterol in baboons differed little by rank, whereas HDL cholesterol was directly related to it [10]. In addition, subordinate baboons had higher resting cortisol levels than did their dominant companions, and hormone levels were inversely associated with HDL cholesterol levels. This correlation matches the proposition of the late Per Bjorntorp that activity of the hypothalamic-pituitary-adrenal axis links psychosocial factors to metabolic syndrome [11]. Further, Sapolsky provided an example of the physiological effects of hierarchy, free of confounding by smoking and alcohol consumption, both of which influence HDL cholesterol levels.

Equally intriguingly, Sapolsky's group recently reviewed the primate literature on social rank differences in cortisol levels [12]. They identified two variables that significantly predicted the ratio of basal levels in subordinate versus dominant animals across seven species. Subordinates exhibited higher relative cortisol levels when they were subjected to higher rates of physical and psychosocial stressors, and when they experienced decreased opportunities for social and kin support. In human males, a high cortisol/testosterone ratio predicted fatal and nonfatal CHD events, but did not predict not all-cause mortality, over 17 years of follow-up [13] .

Since our initial observation of the metabolic syndrome gradient, our work has explored a number of aspects of the direct psychosocial pathway to chronic disease. We showed that metabolic syndrome is linked to adrenocortical function and sympathoadrenal and cardiac autonomic activity [14]. We found that in men, a combination of job control (assessing autonomy and decision latitude at work), health behaviors, and a measure of cardiac autonomic function fully explained the social gradient in prevalence of metabolic syndrome [15]. These studies are as yet cross-sectional, and therefore causality cannot be inferred. Our prospective analyses show that job strain increases the risk of incident general (body mass index) and central obesity, metabolic syndrome, and incident CHD [16–18].

**Box 1. The Power of Mind-Body Interaction: Knockout Stress Triggers Cardiovascular Death among Soccer Fans**

Mortality among adults 45 years or over in Holland on 22 June 1996—the day the Dutch football team was eliminated from the European championship—was compared with the 5 days before and after the match and in the same period in 1995 and 1997. Coronary and stroke mortality was increased in men on the day of the match (relative risk 1.51, 95% confidence interval 1.08–2.09). No clear rise in mortality was observed for women (1.11, confidence interval 0.80–1.56). Among men, approximately 14 excess cardiovascular deaths occurred on the day of the match [19].

Many questions remain. The quest for a universal stress biomarker, the Holy Grail of “stress biology,” may be a vain enterprise, but with respect to obesity, diabetes, and cardiovascular disease, metabolic syndrome proves to be a useful construct. Uncertainty remains over the extent to which direct autonomic and neuroendocrine pathways are important causes in comparison to behavior-mediated explanations. A model involving interaction between autonomic function and behavior may be closer to reality, as the work on sleep disturbance and appetite suggests [20].

Separating out long-term causal sequences is difficult. Controlled stress experiments in the laboratory demonstrate the plausibility of mechanisms linking socioeconomic status to disease risk [21], but translation to long-term randomized trials is problematic. More feasibly with the newly available high-throughput genotyping technology, we plan to harness Mendel's second law—nature's randomization—to compare stress hyper-responders with their more even-tempered counterparts. By comparing the lifelong effects of genotype with observed phenotype, we have the potential to break the confounding intrinsic to conventional epidemiology, to clarify what is cause and what is effect. However this enterprise evolves, there remain the twin tasks of better understanding the reasons why health inequalities continue in rich societies and contributing to efforts to reduce those inequalities.

## Abbreviations

CHD: coronary heart disease
HDL: high-density lipoprotein
LDL: low-density lipoprotein
